# Implementation of minimal invasive gynaecological surgery certification will challenge gynaecologists with new legal and ethical issues

**Published:** 2016-06-27

**Authors:** V Tanos, R Socolov, P Demetriou, M Kyprianou, A Watrelot, Y Van Belle, R Campo

**Affiliations:** Aretaeio Hospital and St Georges Medical School, Nicosia University, Nicosia, Cyprus.; University of Medicine and Pharmacy Gr. T. Popa, Iasi, Romania.; 2, Agias Elenis Street, Stasinos Building, 7th Floor, 1060 Nicosia, Cyprus.; Hôpital NATECIA, 22 Avenue Rockefeller 69008-Lyon-France.; European Academy of Gynaecological Surgery, Diestsevest 43, 3000 Leuven, Belgium.; LIFE, Tiensevest 168, 3000 Leuven, Belgium.

**Keywords:** Diploma, ECRES, endoscopic surgery, ethics, GESEA, insurance companies, medical education, medicolegal

## Abstract

The introduction of a certification / diploma program in Minimal Invasive Surgery (MIS) is expected to improve surgical performance, patient’s safety and outcome. The Gynaecological Endoscopic Surgical Education and Assessment programme (GESEA) and the ESHRE Certification for Reproductive Endoscopic Surgery (ECRES) provides a structured learning path, recognising different pillars of competence. In order to achieve a high level of competence a two steps validation is necessary: (a) the individual should be certified of having the appropriate theoretical knowledge and (b) the endoscopic psychomotor skills before entering in the diploma programme reflecting the surgical competence. The influence of such an educational and credentialing path could improve safety and offer financial benefits to the hospitals, physicians and healthcare authorities. Moreover the medicolegal consequences can be important when a significant amount of surgeons possess the different diplomas. As the programs are becoming universally accessible, recognised as the best scientific standard, included in the continuous medical education (CME) and continuous professional development (CPD), it is expected that a significant number of surgeons will soon accomplish the diploma path. The co-existence and practice of both non-certified and certified surgeons with different degrees of experience is unavoidable. However, it is expected that national health systems (NHS), hospitals and insurance companies will demand and hire doctors with high and specific proficiency to endoscopic surgery. When medico-legal cases are under investigation, the experts should be aware of the limitations that individual experience provides. The court first of all examines and then judges if there is negligence and decides accordingly. However, lack of certification may be considered as negligence by a surgeon operating a case that eventual faces litigation problems. Patients’ safety and objective preoperative counselling are mandatory, directly connected to MIS certification while eliminating any dispute of surgeons’ credibility.

## Introduction

Minimal invasive surgery (MIS) assessment and certification is considered to be the gold standard in assuring that a surgeon has acquired and retained a certain level of knowledge and skills. The European University hospitals that train gynaecologists to perform minimal invasive surgery do not follow a common educational programme neither is endoscopic surgery considered a learning priority element for a residency programme. The system of fellowships for the subspecialty training programme that exists in the USA does not exist in Europe; hence the vast majority of endoscopic surgeons have been self-trained by visiting medical centres with reputation of MIS, or inviting surgeons with experience in the field to their facilities. In addition, most of the endoscopic surgeons participate occasionally in workshops, congresses and hands-on training courses with dry and wetlabs and or animal models. The industry has always been supportive to gynaecologists with special interest to MIS, thus organizing training courses and teaching emerging technologies, equipment and instruments.

The MIS practice on human patients should be regarded with caution. A big number of gynaecologists practicing MIS today did not follow a proper lab and or animal training prior to operate on humans. Today a young gynaecologist interested in practicing laparoscopy and or hysteroscopy should first have excellent theoretical knowledge, training in simulators, exercise in dry and wet lab and animal models. Once if a certification is acquired, he/she could initiate practice on humans under supervision until sufficient experience is gained. The co-existence and practice of both non- certified experienced and certified less experienced gynaecologists is unavoidable, especially during the early stages of certification implementation. When medico-legal cases are under investigation, the experts should be aware of the limitations that individual experience provides. Entry complications are specific, and not always related to surgeons experience however, the conversion of laparoscopic hysterectomy to laparotomy is up to 25% when surgeons are not properly trained (Hebbar et al., 2006).

A Dutch study reported that 18% of malpractice claims related to accidents only were from properly consented patients (Wind et al., 2007). Of course the appropriate consent form derives from the knowledge, skills and experience of the surgeon and for complicated cases an additional consent form should be signed based on the individual case. Proper and objective patient counselling prior to surgery is the result of surgeons’ education and training, of being aware of potential complications and the realistic estimation of treatment rate of success. It is indisputable that CME and CPD is the responsibility of each individual doctor and should be the driving force to excellence.

The broad implementation of certification in minimal invasive surgery (MIS) is expected to improve surgery outcome and increase patients’ safety. At the same time ethical matters and issues of principle will appear in the medico-legal cases especially when the number of certified surgeons will increase. The accessibility and the low cost of MIS certification are crucial factors for implementation among a large number of gynaecologists. It is expected that national health systems, hospitals and insurance companies will demand and hire doctors with high and specific proficiency in endoscopic surgery, due to the fact that the safety and objective preoperative counselling of patients are of utmost importance, while certification of MIS will eliminate any dispute of surgeons’ credibility. In this paper we try to demonstrate that certification in MIS is mandatory, it is one way route with no turning back and an important continuous professional development that every gyne-endoscopic surgeon should follow in order to improve patients’ treatment and safety.

## The importance of professional training in MIS prior to surgery on human beings

In the last 40 years dedicated surgeons like K. Semm, M. Bruhat, C. Sutton and other gynaecologists discovered the benefits of MIS and promoted its implementation among their institutions and colleagues. They learned from each other and initiated various training programmes, mainly by tutor -apprentice operating and training during live surgery. Adopting new instruments and techniques has been the result of the continuous technological advancements while the importance of ergonomy, training and testing only recently gained interest and importance. Actually, in 2007 the Dutch Ministry of Health detected high morbidity and mortality rates after common endoscopic surgery and banned the technique for 2 years. Searching for the reason, lack of training was the most important factor of these results, hence all surgeons should present to the authorities their experience and training background. The Dutch Inspectorate of Healthcare in 2007 (IGZ, 2007) enforced the development of structured competency-based training programmes in general surgery, obstetrics and gynaecology and urology (www.igz.nl, IGZ: Dutch Health Care Inspectorate).

Today it is considered mandatory for a young gynaecologist interested to practice gynaecologic endoscopic surgery (laparoscopy and hysteroscopy) to have excellent basic theoretical knowledge on the subject, to practice training in simulators, dry and wet lab, to train on animal models, to acquire certification and then initiate clinical practice under supervision until adequate experience is gained (Molinas and Campo, 2010).

All gynaecologists should only intervene on patients when they are properly trained and certified. However, the training system is not standardised in curricula, duration and pre-requirements. The Australasian Gynaecologic Endoscopy and Surgery (AGES) evaluate the trainee’s academic and clinical abilities and professional qualities (AGES Society accredited training program, 2013). In Germany every gyne-endoscopist surgeon must perform at least 500 major operations per year to be considered safe enough for practicing surgery. In other European countries there are no additional criteria or requirements for surgeons using MIS, once they have been registered in the specialty of obstetrics and gynaecology. Before any obligatory training is introduced the optimal format should be studied in comparative and prospective studies. A possible agreement on the content, steps and timing of such a training by the academic institutions and professional bodies would facilitate and accelerate such an effort.

The Gynaecological Endoscopic Surgical Education and Assessment (GESEA) programme, provided by ESGE, targets all gynaecologists practice endoscopic surgery to have a common educational platform. The principles of general surgery and specific endoscopic operative skills and techniques and knowledge are included in a structured training programme. The European Academy of Gynaecological Surgery (EAGS) provides courses with validate tests, reassuring effective training for both novice and experienced surgeons. Psychomotor skills such as camera handling, hand-eye coordination and bi-manual instrument handling in the pelvic environment, stitching and knotting are essential credentials for every endoscopic surgeon and direct to safe and good surgery outcome (Campo et al., 2012). The ESHRE (European Society of Human Reproduction and Embryology) special interest group in reproductive surgery provides campus courses in training and testing in reproductive surgery twice a year. Several other training programmes of 3-4 days are also available, aiming to continuous gynaecological endoscopic surgery training with reasonable prices.

## Continuous surgery education and training in gynaecological endoscopy

The interest to improve knowledge and updating on techniques and new equipment and instruments is clearly a responsibility of the surgeon. Patient safety issues arise with the diffusion of a new procedurally- based technology. The loss of tactile input is the major factor in making minimal access techniques difficult to learn. The threshold of 25-30 cases is an arbitrary number of course, which varies according to the operation difficulty and surgeon’s previous experience, however a certain amount of cases are mandatory before surgeons attain proficiency. This proficiency becomes greater as the complexity of the procedures grows, as 90% of injuries are predicted to occur during a surgeon’s first 30 cases (Soot et al., 1999). Surgeons who performed procedures without additional training were 3 times more likely to have at least one complication compared with surgeons who attended additional training (Gibbs et al., 2012). Therefore, when an action is brought to court based on a complication, the above are serious factors for consideration and may alter the court’s decision for or against the doctor.

**Fig. 1:**
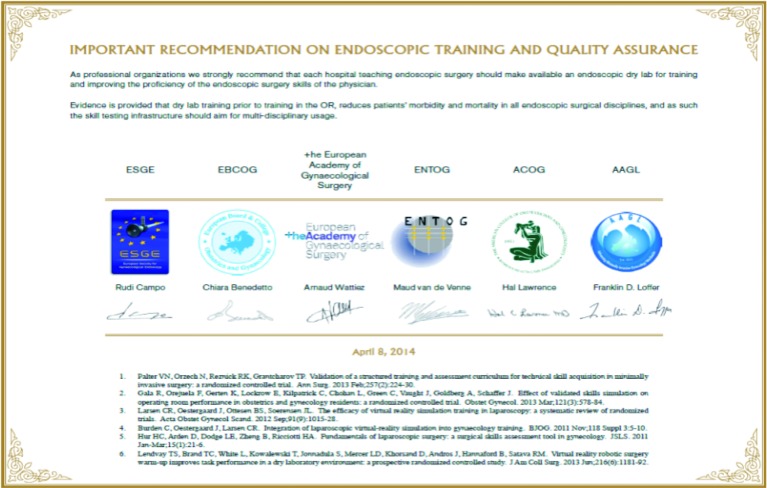
— Recommendation on endoscopic training and quality assurance signed and agreed by 6 professional societies in Europe and USA.

However, once a surgeon has been through training and testing and is certified, his position in court might have a favourable perspective. In July 2014 an important recommendation on endoscopic training and quality assurance was signed and agreed by professional societies in Europe and USA {Joint statement of European Society for Gynecologic Endoscopy, (ESGE), European Board and College for Obstetrics and Gynecology (EBCOG), European Academy of Gynecologic Surgery (EAGS), European Network of Trainees in Obstetrics and Gynecology (ENTOG), American College of Obstetrics and Gynecology (ACOG) and the American Association for Gynecologic Laparoscopy (AAGL) (Fig. 1). (www.esge.org/images/pdf/Joint)

They strongly recommend that each hospital teaching endoscopic surgery should make available an endoscopic dry lab for training and improving the proficiency of the endoscopic surgery skills of the physician. Evidence is provided that dry lab training prior to practice in the OR, reduces patients’ morbidity and mortality in all endoscopic surgical disciplines and as such the skill testing infrastructure should aim for multi-disciplinary usage (Molinas and Campo, 2010; Palter et al., 2013). Such statements and opinions might be brought before the court in a medical malpractice suit so as to indicate the grade of professionalism and responsibility, as well as the measures taken by the hospital management. Of the approximately 2000 accredited CME programs, about two thirds participate in quality-improvement initiatives within their health systems and institutions. Hospital and health system leaders report that investment in CME has helped them improve physician performance, patient outcomes and care coordination (McMahon, 2016).

## Training Protocols in MIS (Minimal Invasive Surgery)

A surgeon of MIS needs a theoretical knowledge of anatomy, pathology, treatment options, surgical techniques and laparoscopic psychomotor skills (LPS) correct camera handling, hand-eye coordination, and bimanual coordination. The gaining of surgical competence is a continuous learning process, demanding one-to-one learning with a highly skilled surgeon. Continuous practice and experience are necessary. In LPS the assistance of a highly skilled surgeon is less important than training of exercises and the gained ability remains over a long period of time.

It is difficult to acquire laparoscopic skills during surgical training in the operating room as the apprentice first observes, then helps and finally operates under guidance. The apprentice-tutor model, which was historically used to teach open surgery, cannot simply be transferred to laparoscopy. Achieving proficiency in both general surgical skills and specific LPS using this model seems not only impossible but also ethically unacceptable, due to the increased operating time and higher complication rate. Furthermore, the limitation of resident work hours, the limited number of skilled tutors available, and long learning curves reported (the high number of procedures needed to achieve proficiency) make this model unsuitable as the only method for training in MIS (Aggawal et al., 2006; Ascher-Walsh and Capes, 2007; Simons et al., 1995; Ghomi et al., 2007). Once endoscopic surgery is performed without the required skills and expertise, patients are exposed to unnecessary high risk for serious complications (van der Wal, 2014). Hence, it is essential that a part of the training takes place outside the OR. Several training modalities have been developed as virtual reality, box trainers, animal models and others as shown in table I. Although animal models are most suitable to simulate real-life situations, availability of these models is limited due to ethical concerns and high costs. The inanimate models have the advantage of allowing longer training periods, ensuring full LPS acquisition and not only exposure to specific MIS tasks. Trainer boxes are relatively cheap and accessible (Katz, 2006), whereas virtual reality models provide an objective evaluation of the learning process (Gor et al., 2003) but are still very expensive, making a broad implementation difficult. Both trainer boxes and virtual reality models are equally effective for acquiring laparoscopic skills (Munz et al., 2004). However, most of these models are not validated, not used in a standardized way, and not easily available. Furthermore, validated, well-structured, and generally accepted training and certification programs, including pre-clinical training, are not universally implemented although there is an urgent need for a standardized and structured training, certification and diploma program in endoscopic surgery (Campo et al., 2012a, 2012b).

**Table I:** — The sequence of training protocols and certification process.

1. Theoretical knowledge (books, Journals, Internet enduring materials, conferences, courses, regularly scheduled series and other live sessions)
2. Grand ward rounds
3. Hands on training (dry lab psychomotor skills)
*Certification of psychomotor skills*
4. Hands on training (simulators, wet lab, animal models, animals, fresh cadavers)
5. Operating Room training, under supervision for easy cases
*Diploma in MIS as Bachelor*
6. Operating Room training, under supervision for difficult cases
7. Independent activity in MIS
*Diploma as expert/master*

The GESEA programme is an integrated educational and training program in gynaecological endoscopic surgery, differentiating the different skills in different learning and accreditation paths. It recognizes that to achieve a level of competence, a two-step validation is necessary, first the individual should be certified of having the appropriate theoretical knowledge and endoscopic psychomotor skills before entering the diploma programme which reflects the surgical competence. The sequence and steps of training and accreditation to follow are presented in Table I.

## Certification in Gynaecological MIS

Certification in Gynaecological MIS examines the learning path and the level of the theoretical knowledge, the psychomotor and surgical skills acquired, recognizing different pillars of competence. In order to achieve a high level of competence, a two-step validation is necessary. The individual is certified of having the appropriate endoscopic surgery theoretical knowledge and psychomotor skills reflecting the surgical competence before entering in the diploma programme. The GESEA programme ensures that board certification provides surgeons with recognizable skill levels and standards of excellence. Assessment and certification is considered to be the gold standard in assuring that a surgeon has acquired and retained a certain level of knowledge and skills. The GESEA programme provides in a Level 1 Diploma of ESGE Bachelor in Endoscopy, a Level 2 Diploma of ESGE Minimal Invasive Gynaecological Surgeon (MIGS) or the third level or Master level in which 2 diplomas can be achieved the Master of Hysteroscopy and or the Laparoscopic Pelvic Surgeon.

The ESHRE Certification for Reproductive Endoscopic Surgery (ECRES) provides the Primary Level for Reproductive Surgeon and a higher level of Master in Reproductive Surgery. The broad implementation of both certification systems will improve the treatment results and increase patients’ safety. The syllabus of GESEA and ECRES includes e-learning structured modules on gynaecological endoscopic surgery accompanied by MCQs, validated exercises for training and testing, and a final examination of knowledge at the highest scientific level. The ECRES syllabus also includes an electronic surgery logbook and review of 15 surgeries by unedited videos. Both certification programmes are strict and demanding. Hard preparatory work on hand skills, experience in operating room, exposure and good understanding of the recent developments of MIS are mandatory in order for someone to pass all the examination steps and obtain the certification in gynaecological endoscopic surgery. Moreover it encourages young gynaecologists interested in practicing MIS to train well and gain proficiency prior to practicing on humans.

The influence of such an educational and credentialing path could improve the safety and offer financial benefits to the hospitals, physicians and healthcare authorities. Moreover the medico-legal consequences can be important when a significant amount of surgeons possess the different diplomas. As the programs are becoming universally accessible, recognised as the best scientific standard, included in the continuous medical education (CME) and professional development (CPD), we expect that a significant number of surgeons will soon accomplish the diploma path. At the same time ethical and legal matters might be raised for those gynaecologists who will fail or not even apply for a certification, but continue to practice minimal invasive surgery. When surgeons who underwent a recognizable education and testing are called to defend their position for a mishappening on an ethical or legal level, they will have an additional strong argument supporting their competence to practice, especially when a difficult operation is performed. Training and certification on gynaecological endoscopic surgery will play a pivotal role in the near future when medico-legal problems occur. Gynaecological Societies like the ESGE and ESHRE will be inevitably used as authorities of reference and might be also called to court to defend and justify the abilities and performance of their certified surgeons’ members.

## Medico-legal issues as a consequence of endoscopic surgery certification availability

Knowledge, skill acquisition, ethical matters as well as issues of principle will appear in the medicolegal cases once the number of certified surgeons will increase. In parallel National Health Systems and hospitals will demand and hire doctors with high and specific proficiency to endoscopic surgery. In Germany every endoscopist surgeon must perform at least 500 major operations in order to be eligible to continue his surgery activities because under these circumstances patients 'safety is secured. The alleged great advantage of endoscopic surgery either in hysteroscopy or in laparoscopy to observe details that you cannot watch by laparotomy opens another ethical and legal issue in those cases where complications occurred after laparotomy. We must admit that studies comparing complications between gynaecological laparoscopic surgery and laparotomy do not have significant differences (Chapron et al., 2002; Kreiker et al., 2004).

Continuous medical and surgical education should involve hot issues like new procedures and reassurance of how to manage complications. The risk of complications depends not only on the surgeon’s experience but most important on the surgeon’s knowledge and current practice. This emphasizes the need for learning the anatomy, strict follow up on surgery rules, learning the suturing techniques, the principles of energies and other important new techniques and procedures. In the unwished event of a medico-legal case, the experts should be aware of the limitations that individual experience provides (Twijnstra et al., 2012). The patients should be well informed and consent prior to operation; the importance of good equipment, the gynaecological facilities and environment within the hospitals should be all part of the legal solution. There are different protocols that deal with risk management in surgery, and they could be adapted to gynaecologic endoscopy surgery (Haynes et al., 2009). But again this has to be a part of authority position, as it should be implemented both in daily life and especially in those cases that are at the high risk for medico-legal situation.

## Legal aspects and opinion

The implementation of the MIS certifications aims to reduce complications and improve surgery outcome. Extensive training is needed to pass the psychomotor skill examinations and deep knowledge of anatomy, endoscopic surgery techniques and gynaecological pathologies required to pass difficult theoretical examinations. Nevertheless, it is not established that a surgeon who does not opt to undergo the training for the certification will be in a lesser position to perform such surgery. In situations where a gynaecologist faces court proceedings for an alleged malpractice the court’s decision will be based upon the standard of care expected from a doctor of his skill and knowledge.

The test for this standard of care, as established in the English case of Bolam v Friern Hospital Management Committee [1957] 1 WLR 582, is that of an ordinary skilled man exercising and professing to have that special skill. This means that the gynaecologist in question is not required to possess the highest expert skill, but to act in accordance with the accepted medical practice in respect to acts or omissions made by a professional with his skills and knowledge. When considering the above it appears that, from a legal point of view, it is not safe to state that it is favourable for a gynaecologist to be experienced though uncertified on MIS, than to be a certified relatively young gynaecologist and vice versa. Each doctor, either certified or not, will be judged based on his personal skills and knowledge while exercising his her profession. When determining the accepted medical practice, the court will hear the testimony of expert witnesses and judge on whether the act or omission in question is in conformity with a responsible body of opinion within the profession. An expert witness is someone who by virtue of education, training, skill or experience is believed to have expertise and specialised knowledge in a particular subject beyond that of the average person. In other words, a person can be considered as an expert witness based on either his academic qualifications or his experience. Therefore, an uncertified gynaecologist with experience on the practise of MIS may be deemed as an expert witness, regardless of whether he has acquired a certification on MIS. A gynaecologist with lesser experience but certified, may be considered as an expert witness for the purposes of establishing the accepted medical practice in a malpractice suit. Nevertheless, the opinion expressed by the expert witnesses must have a logic basis and eventually convince the court that their opinion is the correct one and thus constitutes the accepted medical practice.

Regardless of the gynaecologist status and whether or not is certified on MIS, there is no legal obligation on the doctor to disclose to patients this information. However, the doctor may choose to disclose this fact on an ethical point of view, if he/she is of the opinion that the said information may alter the patient’s decision on the desired surgeon or on undergoing a surgery. Furthermore, most of the European countries Medical Associations and Ministries of Health are not legally obliged to inform the public about the existence of gynaecologists who are certified to perform MIS. Nevertheless, the Medical Associations and especially the ObGyn Societies must promote the continuous training and scientific development of its members and in doing so they should notify its members on new techniques and skills that they may acquire through further training. If medical institutions face court proceedings based on the lack of certification on MIS among its doctors then the insurance companies may pressure the institutions to hire surgeons certified on MIS so as to limit the liability of the hospital on a malpractice suit. However, this depends solely on the court’s findings on these matters and whether or not it will hold a hospital liable for not providing a certified gynaecologist. On this note, medical institutions may demand their doctors to be certified on MIS so as to escape liability.

## Conclusive remarks

This is clearly an opinion paper and the text should not be misunderstood as some official guideline. According to dictionary, an expert is a person with extensive knowledge or ability based on research, experience or occupation in a particular area of study. Lawyers depict doctors and introduce them in front of courts as experts. In daily practice when a medical case appears in court, the expert is usually a doctor who declares himself an expert or the advocate identifies his expertise according to the number of publications or years of experience in the profession. Often these expert doctors are heads of the departments in Obstetrics and Gynaecology. However the first thing that the court must establish is negligence. The strong argument about having or not the certification in endoscopic surgery is whether lack of certification comprises negligence by the surgeon who performs endoscopic surgery. Also having the certification is a proof of surgeon’s competence and it is difficult to be disputed since it is independently examined and granted. In the case of aviation industry even experienced pilots undergo simulation training and testing and certification of their acquired skills once they have to fly another model of airplane. Within this context novice as well as experienced gynaecologists should undergo training and testing and certification of endoscopic surgery of their knowledge and handskills at least once in their life. Of course certification is not for life and it better should be renewed every 5 years. Certification definitely provides an important step to secure patients’ safety and offers better operative results while minimizing complications.
